# Enhancement of adriamycin-induced killing after delayed plating of plateau-phase V79-cells.

**DOI:** 10.1038/bjc.1986.169

**Published:** 1986-08

**Authors:** G. Iliakis, M. Nusse, J. Egner

## Abstract

Unfed plateau-phase cultures of Chinese hamster V79-cells were treated for 1 h with various amounts of adriamycin in the range between 0 and 10 micrograms ml-1 and subsequently either immediately trypsinized and plated to assay for survival, or reincubated in medium collected from replicate plateau-phase cultures and returned to the incubator for various periods of time before plating. Significantly less killing was observed, for the same adriamycin dose, in cells treated in the plateau-phase and plated immediately thereafter as compared to cells treated while actively growing. When cell trypsinization and plating was delayed for up to 22 h, a significant increase in killing was observed, and the survival curve obtained approached that observed after treatment with adriamycin of growing cells. Initially almost exponential kinetics were observed for this potentiation of adriamycin-induced cell killing with a t37 of approximately 2 h. Cell survival was still decreasing after 22 h of post-treatment incubation in the plateau phase, with no clear indication for approaching a plateau. However, longer incubations, to establish a plateau, were not possible due to degeneration of the cultures. Flow cytometry measurements of the intracellular adriamycin content showed only a small difference between exponentially growing and plateau-phase cells despite the significant differences in the number of cells per culture at the time of treatment. The rate at which adriamycin-related fluorescence decayed after adriamycin treatment was slightly higher for cells trypsinized and exposed to fresh medium than for cells kept in the plateau-phase. The results indicate the importance of the physiological state and the post-treatment incubation conditions of cells for the final effect of adriamycin on survival.


*Br. J. Cancer* (1986), **54**, 245–249

### Enhancement of adriamycin-induced killing after delayed plating of plateau-phase V79-cells

G. Iliakis, M. Nusse* & J. Egner*

*Thomas Jefferson University Hospital, Department of Radiation Therapy and Nuclear Medicine, Laboratory of Experimental Radiation Oncology, Philadelphia, PA 19107, USA.*

**Summary** Unfed plateau-phase cultures of Chinese hamster V79-cells were treated for 1 h with various amounts of adriamycin in the range between 0 and $10\,\mu g\,ml^{-1}$ and subsequently either immediately trypsinized and plated to assay for survival, or reincubated in medium collected from replicate plateau-phase cultures and returned to the incubator for various periods of time before plating. Significantly less killing was observed, for the same adriamycin dose, in cells treated in the plateau-phase and plated immediately thereafter as compared to cells treated while actively growing. When cell trypsinization and plating was delayed for up to 22 h, a significant increase in killing was observed, and the survival curve obtained approached that observed after treatment with adriamycin of growing cells. Initially almost exponential kinetics were observed for this potentiation of adriamycin-induced cell killing with a $t_{37}$ of $\sim 2$ h. Cell survival was still decreasing after 22 h of post-treatment incubation in the plateau phase, with no clear indication for approaching a plateau. However, longer incubations, to establish a plateau, were not possible due to degeneration of the cultures. Flow cytometry measurements of the intracellular adriamycin content showed only a small difference between exponentially growing and plateau-phase cells despite the significant differences in the number of cells per culture at the time of treatment. The rate at which adriamycin-related fluorescence decayed after adriamycin treatment was slightly higher for cells trypsinized and exposed to fresh medium than for cells kept in the plateau-phase. The results indicate the importance of the physiological state and the post-treatment incubation conditions of cells for the final effect of adriamycin on survival.

Much higher doses of adriamycin are usually required to achieve the same extent of killing in plateau-phase than in actively growing cell populations (Barranco, 1975; Barranco & Noval, 1974; Twentyman & Bleehen, 1975; Sutherland, *et al.*, 1979; Martin & McNally, 1980; Chambers *et al.*, 1984). This decrease in the adriamycin killing effect was partly attributed to the differences pertaining to the numbers of cells per culture during treatment in exponentially growing *vs.* plateau-phase cells, and it was found to decrease or disappear when cell survival was plotted against the amount of adriamycin per cell (Sutherland *et al.*, 1979; Chambers *et al.*, 1984). Examination of the intracellularly accumulated amount of adriamycin, however, indicated a significantly lower killing effect in plateau-phase cells for similar intracellular drug accumulation (Chambers *et al.*, 1984), suggesting that the distinct physiological state of cells in the plateau-phase may significantly affect adriamycin-induced cell killing (Barranco 1975; Twentyman & Bleehen, 1975; Martin & McNally, 1980). Plateau-phase cultures have been proposed to be an *in vitro* model describing a tumour more closely than actively growing cultures (Hahn & Little, 1972), and cells at this physiological condition were found to have increased ability to repair radiation-induced damage, if a few hours are allowed to elapse between irradiation and subculture (delayed plating, DP) (Hahn & Little, 1972; Iliakis & Pohlit, 1979). A similar repair activity was also observed after irradiation of experimental tumours *in vivo* (Hahn *et al.*, 1974).

In this work we report the survival modifications observed after exposure of plateau-phase cells to adriamycin followed by either immediate (IP) or delayed (DP) plating. Delayed-plating protocols mimic the tumour situation more closely than immediate-plating protocols since *in vivo* cells are not transferred to a 'new' environment after treatment. Contrary to the results obtained under similar conditions after exposure to ionizing radiation, delayed plating of cells resulted in a dramatic potentiation of the adriamycin mediated killing, and caused plateau-phase cells to respond to treatment similarly to actively growing cells.

### Materials and methods

For the experiments, Chinese hamster V79-cells

*Present address: Gessellschaft fuer Strahlen-und Umweltforschung Paul-Ehrlich-Str.20, 6000 Frankfurt/Main 70, FRG.

Correspondence: G. Iliakis.

Received 11 December 1985; and in revised form, 11 March 1986.

© The Macmillan Press Ltd., 1986

(S171) were used. Details about their origin and growth conditions have been published (Iliakis, 1985). Briefly, cells grew at 37°C in a humidified atmosphere of 5% $CO_2$ in MEM supplemented with 15% foetal calf serum. Cells were subcultured every 2 days starting at concentrations of $10^6$ cells/25 $cm^2$ tissue culture flask (20 ml MEM). For the experiments, cells from these cultures were plated at a concentration of $4 \times 10^4$ cells/dish (50 mm, 5 ml MEM per dish) and were used 4 days later. At this time, cells had reached a plateau-phase with more than 80% of the cells (compared to ~30% in growing cell populations) accumulated in a phase showing a DNA content equivalent to that of $G_1$ cells (flow cytometry measurements). The cultures were never refed (unfed plateau-phase cultures) and the cessation of growth is attributed to the exhaustion of one or more of the nutrients from the medium. The pH of the cells in the plateau-phase of growth was $7.1 \pm 0.1$. The doubling time of the cultures in the exponential phase was ~9 h and the concentration reached in the plateau-phase 6–8 $\times 10^6$ cells/dish. There was no cell detachment observed in the cultures at this state.

Adriamycin (Sigma) was given to the cells from a 1 mg $ml^{-1}$ water solution and was allowed to act for 1 h (37°C). After this time period, the medium was removed from the dishes and cells were rinsed twice with phosphate buffered saline. Subsequently, one set was returned to the incubator after addition of 5 ml medium gained from replicate plateau-phase cultures after filtration to remove viable cells, and one set was trypsinized and plated. Trypsinization and plating of the set of cultures that were returned to pre-treatment conditions were carried out at various times after treatment depending on the experimental protocol. The trypsinization was performed with 0.5% trypsin and 0.2% EDTA for 15 min, but it was shortened to 5 min (enough to cause complete detachment of the cells from the dishes) when flow cytometry measurements of intra-cellular, adriamycin-related fluorescence were to follow. Cells were plated to form colonies in two 60 mm tissue culture dishes and were incubated at 37°C for 6–7 days. Twenty-five to 200 colonies were counted per dish, the standard errors of counting in the estimation of cell survival thus being between 7% and 14%. Curves were fitted to the data points by eye. All results reported have been confirmed in at least two independent experiments.

For the measurements of intracellular adriamycin concentration, flow cytometry was used (Krishan & Ganapathi, 1980; Durand & Olive, 1981). It has been shown that this method gives results similar to those obtained by fluorimetry (Chambers et al., 1984). After treatment with adriamycin, and according to the needs of each particular experiment, cells were trypsinized or simply collected by pipetting, and a single cell suspension was run through a flow cytometer (cyto-fluorograph, Ortho). Cells were suspended in PBS and were kept after collection and before measurement on ice. Excitation of adriamycin was at 448 nm (Spectra Physics laser model 2025-05 operating at 1500 mW) and fluorescence emission was collected at above 550 nm using a Schott long pass filter (OG550). Each measurement resulted in a histogram storing cells according to their relative adriamycin related fluorescence in 1024 channels. The coefficient of variation of these histograms was between 10–20% and similarly for exponentially growing and plateau-phase cells. Only a comparison of the relative fluorescence intensities of cells in various conditions and physiological states was attempted, and a calibration reverting these values to absolute adriamycin concentration was not performed. The results are shown as mean channel number for the fluorescence intensity *vs.* extra-cellular adriamycin concentration or time.

### Results

In Figure 1 (panel A) the survival curves of cells are shown obtained after exposure to adriamycin of plateau-phase cells for 1 h. Cells were plated either immediately after treatment (IP) or after a 22 h incubation under plateau-phase conditions (DP). A biphasic response was observed for cells plated immediately after irradiation, the terminal part of the survival curve showing a Do = 10.8 $\mu g\,ml^{-1}$. The shape of the survival curve was found to depend upon the age of the culture, late plateau-phase cultures (results not shown) showing essentially a purely exponential response (with a Do similar to that observed for the terminal part of the survival curve (IP) in Figure 1). Plateau-phase cells appear thus to be significantly more resistant to adriamycin as compared to actively growing cells, whose response is shown for comparison in Figure 1 by the dotted line (Do = 0.135 $\mu g\,ml^{-1}$; results obtained from Iliakis *et al.*, in preparation), for cells plated immediately after treatment; delayed plating of growing cells does not significantly affect survival. However, a dramatic increase in killing was observed when the adriamycin treated cells were returned to plateau-phase conditions for 22 h before plating. The response of cells under these conditions (Do = 0.6 $\mu g\,ml^{-1}$) approached that of actively growing cells.

The kinetics of the potentiation of killing observed after delayed-plating of adriamycin treated plateau-phase cells are shown in panel B of Figure 1 for 1 h treatment with 4 and 10 $\mu g\,ml^{-1}$ adriamycin followed by incubation in the plateau-

**Figure 1** Panel A: Survival curves of cells exposed to various doses of adriamycin for 1 h in the plateau-phase of growth, trypsinized and plated either immediately therafter (IP) or after a 22 h incubation in the plateau-phase (DP). The dotted line shows the survival curve obtained with actively growing cells.

Panel B: Cell survival as a function of time under plateau-phase conditions following a 1 h treatment with the indicated doses of adriamycin.

phase for up to 24 h. Due to degradation of the treated cultures at later times, longer incubations were not carried out. The plating efficiency was not significantly affected ($<10\%$ drop) at this time. A nearly exponential decrease in cell survival was observed after treatment at either of the concentrations used ($t_{37}=2$ h). Although a slower decrease in survival became apparent after $\sim$10 h, a plateau was not yet reached after 24 h of incubation. It is assumed, therefore, that the effect had not yet reached its maximum at this point. Similar results were obtained in three more experiments performed but some variation was observed in the survival levels reached after a 20–24 h incubation in C-med.

In an attempt to correlate the observed effect on cell survival with the intracellular adriamycin levels, actively growing cells ($10^6$/dish) were treated with various amounts of adriamycin, and the adriamycin-related fluorescence was measured by flow cytometry. The results obtained are shown in Figure 2A, where the mean channel $\bar{N}$ for the emitted fluorescence is plotted against the adriamycin concentration. Depsite the large differences in the number of cells, the fluorescence intensities obtained from actively growing and plateau-phase cells were only slightly different.

In order to test whether the potentiation of killing observed after delayed plating of plateau-phase cells was due to a faster clearance of adriamycin from the cells subcultured and incubated in fresh medium after treatment, a set of dishes was treated for 1 h with 10 $\mu$g ml$^{-1}$ adriamycin. Half of the dishes were returned after treatment to plateau-phase conditions, whereas the second half was trypsinized and replated ($3 \times 10^6$ cells/dish) in fresh medium. At various times thereafter, cells were collected and measured for residual, adriamycin-related fluorescence. The results obtained are shown in Figure 2B. A slightly faster decrease of fluorescence was observed for cells trypsinized and plated in fresh medium after treatment. Three hours after treatment, cells in fresh medium showed 40% less fluorescence than cells kept in the plateau-phase, but smaller differences were observed in other experiments.

### Discussion

The results presented in the previous section indicate that plateau-phase V79-cells are more resistant to adriamycin than actively growing cells, a result that cannot be entirely explained by the lower intracellular drug accumulation observed (Figure 2). Similar results were also obtained for Chinese hamster ovary cells (Barranco & Novak, 1974; Barranco, 1975) and EMT6 cells (Twentyman &

**Figure 2** Panel A: Adriamycin-related fluorescence intensity given as a mean channel $\bar{N}$ as a function of the adriamycin concentration (1 h treatment), for actively growing (log-phase) and plateau-phase cells.

Panel B: Decay of adriamycin-related fluoresence intensity following a 1 h treatment with $10\,\mu g\,ml^{-1}$ adriamycin, in cells that have been kept in plateau-phase (C-med) or subcultured in fresh medium (F-med).

Bleehen, 1975; Sutherland *et al.*, 1979). The survival curves obtained (Figure 1) were biphasic indicating the presence of subpopulation of cells with differential sensitivity to adriamycin. Actively or slowly growing cells present in plateau-phase cultures might be the sensitive and resting cells the resistant subpopulations, a hypothesis also supported by the high sensitivity to adriamycin of growing cells and the nearly exponential shape of the survival curve of late – and therefore containing less growing cells – plateau-phase cultures.

Although no difference in the sensitivity to adriamycin of exponentially and plateau-phase cells was found when the results were plotted as survival *vs.* amount of adriamycin per cell (Sutherland *et al.*, 1979; Chambers *et al.*, 1984), plateau-phase cells were found to be significantly more resistant to adriamycin than actively growing cells when cell survival was plotted against intracellularly measured adriamycin content (Chambers *et al.*, 1984). In fact, plateau-phase cells were found to have a higher ability to accumulate adriamycin. Our results also show similar tendencies, indicating a higher accumulation of adriamycin by plateau-phase cells, when plotted against the amount of adriamycin per $10^6$ cells (see Figure 2 and consider the cell numbers at the time of the treatment). These findings suggest that not only the intracellular concentration of adriamycin but also other physiological-state-dependent factors may modify the drug cytotoxicity probably by modifying its interaction with the sensitive target(s).

The potentiation of killing observed after delayed plating contrasts with observations made under similar conditions after exposure of mammalian cells to ionizing radiations (e.g., Han & Little, 1972; Iliakis & Pohlit, 1979). Although this effect could be partly explained by the lower rate of intracellular drug clearance in cells kept in the plateau-phase as indicated in Figure 2, it is unlikely that this rather small difference in the clearance rate could entirely account for such a dramatic potentiation of killing. It is possible that modifications in the metabolic state of the cells initiated after their incubation in fresh medium affect, in a time dependent way, the pattern of interaction of the sensitive target(s) with adriamycin, thus, changing the cellular response to the drug. Furthermore, other factors such as progression of cells through the cycle, cell-to-cell contact, drug release from the cells into the medium and reabsorption by the survivors may result in the potentiation of killing observed. The influence of these parameters on the observed potentiation of killing, as well as the intracellular distribution of adriamycin in plateau-phase cells incubated in fresh or conditioned medium are presently under study.

It is not known whether the observed potentiation of adriamycin killing is a phenomenon of general validity observed in many different systems, or a peculiarity of the cell line and the conditions used. Hahn *et al.* (1979) reported no modification in cell survival after delayed plating of refed plateau-phase cultures of Chinese hamster cells, but the authors do not specify the time interval examined and the conditions applied; it is possible that refed plateau-phase cultures respond somewhat differently to delayed plating after adriamycin exposure than unfed plateau-phase cultures. We are presently investigating this possibility.

The authors are greatly indebted to Professor W. Pohlit, for his generous support of the present work. Special thanks go to Ms S. Bobyock for preparing the artwork, and Mss Douthart and Lazar for typing the manuscript.

### References

BARRANCO, S.C. (1975). Review of the survival and cell kinetics effects of adriamycin (NCS-123127) on mammalian cells. *Cancer Chemotherapy Rep.*, **6**, 147.

BARRABCO, S.C. & NOVAK, I.K. (1974). Survival responses of dividing and non-dividing mammalian cells after treatment with hydroxyurea, arabinosylcytosine or adriamycin. *Cancer Res.*, **34**, 1616.

CHAMBERS, S.H., BLEEHEN, N.M. & WATSON, J.V. (1984). Effect of cell density of intracellular adriamycin concentration and cytotoxicity in exponential and plateau-phase EMT6 cells. *Br. J. Cancer* **49**, 301.

DURAND, R.E. and OLIVE, P.L. (1981). Flow cytometry studies of intracellular adriamycin in single cells *in vitro*. *Cancer Res.*, **41**, 3489.

HAHN, G.M. & LITTLE, J.B. (1972). Plateau-phase cultures of mammalian cells. An *in vitro* model for human cancer. *Curr. Top. Radiat. Res.*, **Q8**, 39.

HAHN, G.M., ROCKWELL, S., KALLMAN, F., GORDON, L.F. & FRINDEL, E. (1974). Repair of potentially lethal damage *in vivo* in solid tumor cells after X-irradiation. *Cancer Res.*, **34**, 351.

HAHN, G.M., BRAUN, J. & HAR-KEDAR, I. (1975). Thermochemotherapy: Synergism between hyperthermia (42–43°C) and adriamycin (or bleomycin) in mammalian cell inactivation. *Proc. Natl Acad. Sci. (USA)*, **72**, 937.

ILIAKIS, G. & POHLIT, W. (1979). Quantitative aspects of repair of potentially lethal damage in mammalian cells. *Int. J. Radiat. Biol.*, **36**, 649.

ILIAKIS, G. (1985). Evidence for the induction of two types of potentially lethal damage after exposure of plateau-phase Chinese hamster V79-cells to X-rays. *Radiat. Environ. Biophys.*, **24**, 185.

KRISHAN, A. & GANAPATHI, R. (1980). Laser flow cytometric studies on the intracellular fluoresence of antracyclines. *Cancer Res.*, **40**, 3895.

MARTIN, W.M.C. & McNALLY, N.J. (1980). Cytotoxicity of adriamycin to tumor cells *in vivo* and *in vitro*. *Br. J. Cancer*, **42**, 881.

TWENTYMAN, P.R. & BLEEHEN, N.N. (1975). Changes in sensitivity to cytotoxic agents occurring during the life history of monolayer cultures of a mouse tumor cell line. *Br. J. Cancer*, **31**, 417.

SUTHERLAND, R.M., EDDY, H.A., BAREHAM, B., REICH, K. & VANANTWERP, D. (1979). Resistance to adriamycin in multicellular spheroids. *Int. J. Radiat. Oncol. Biol. Phys.*, **5**, 1225.


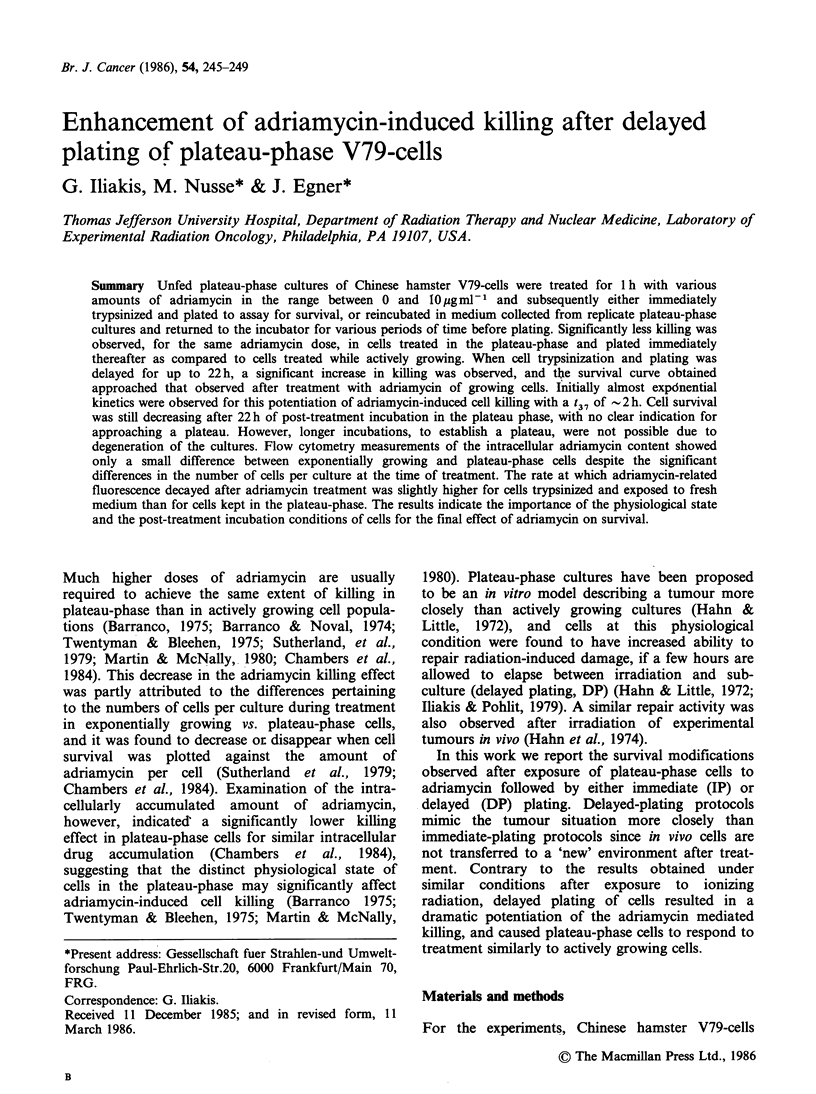

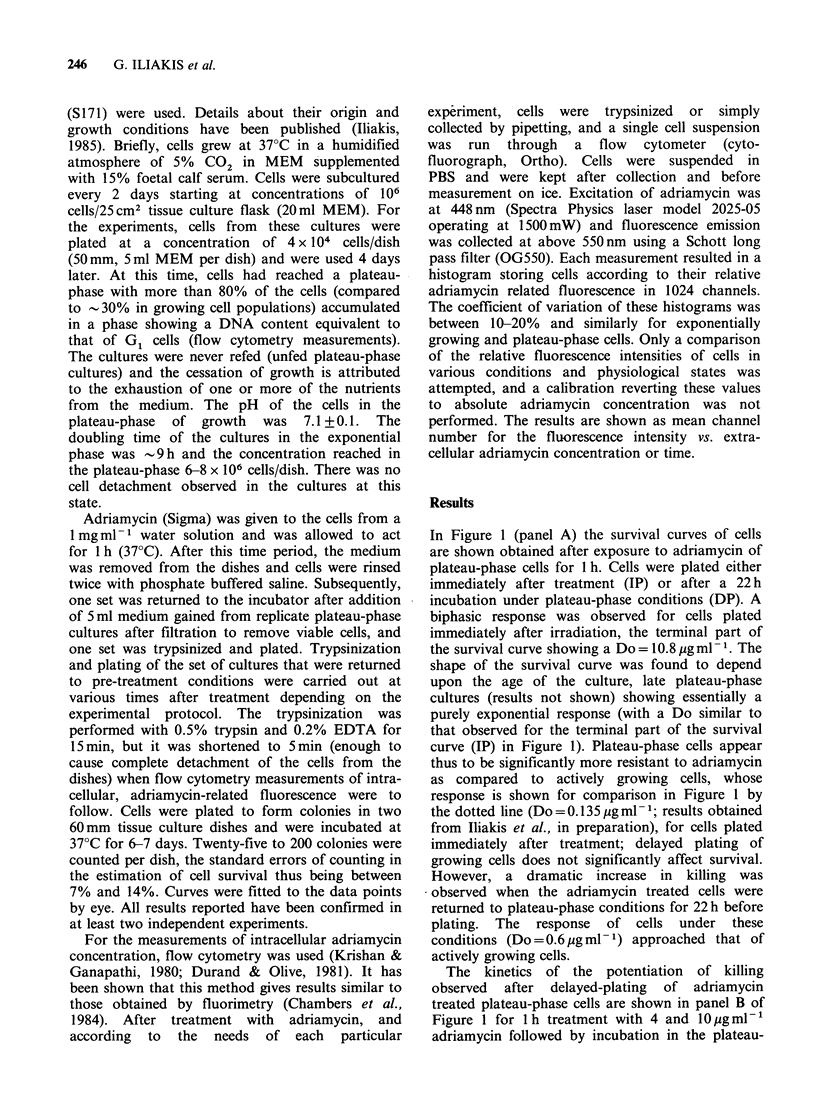

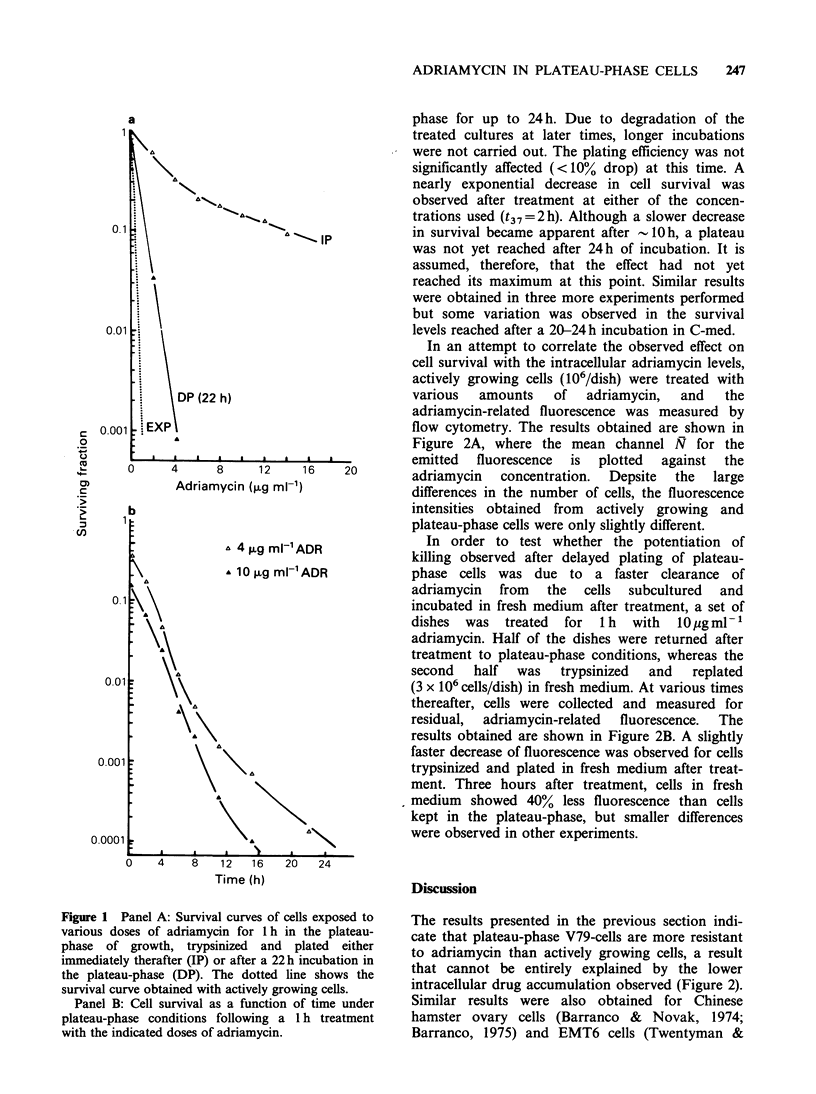

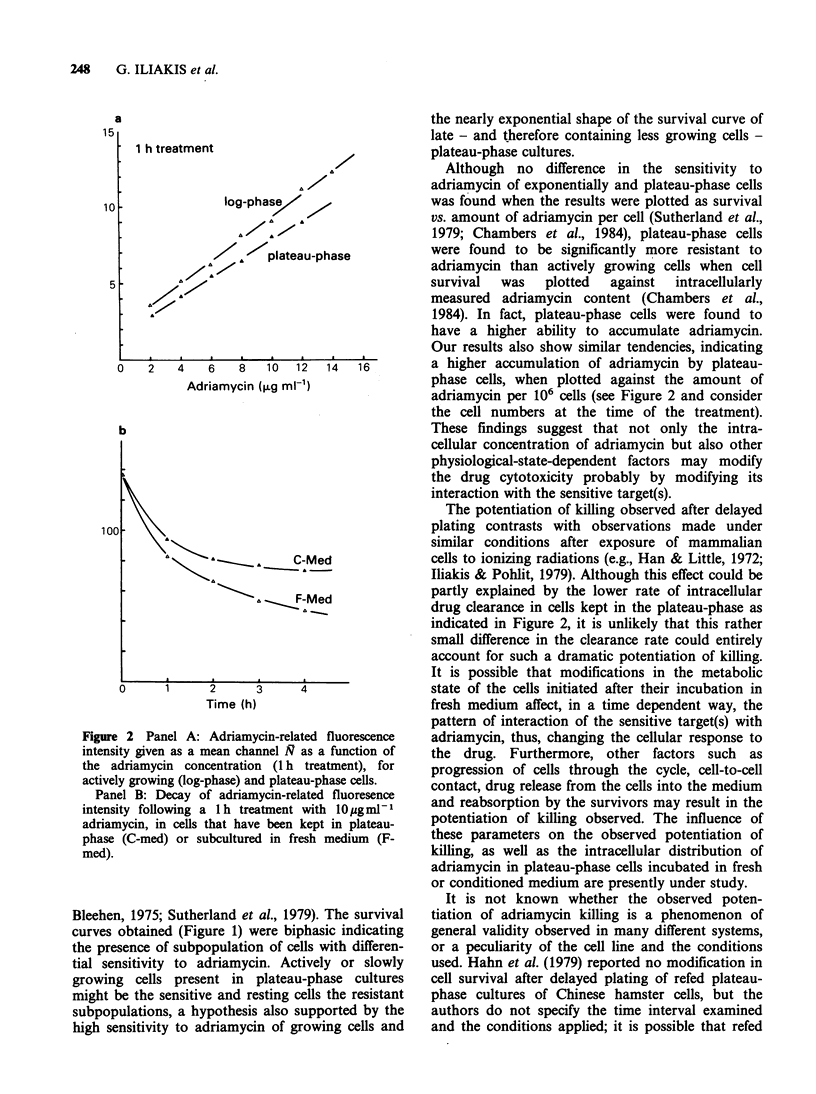

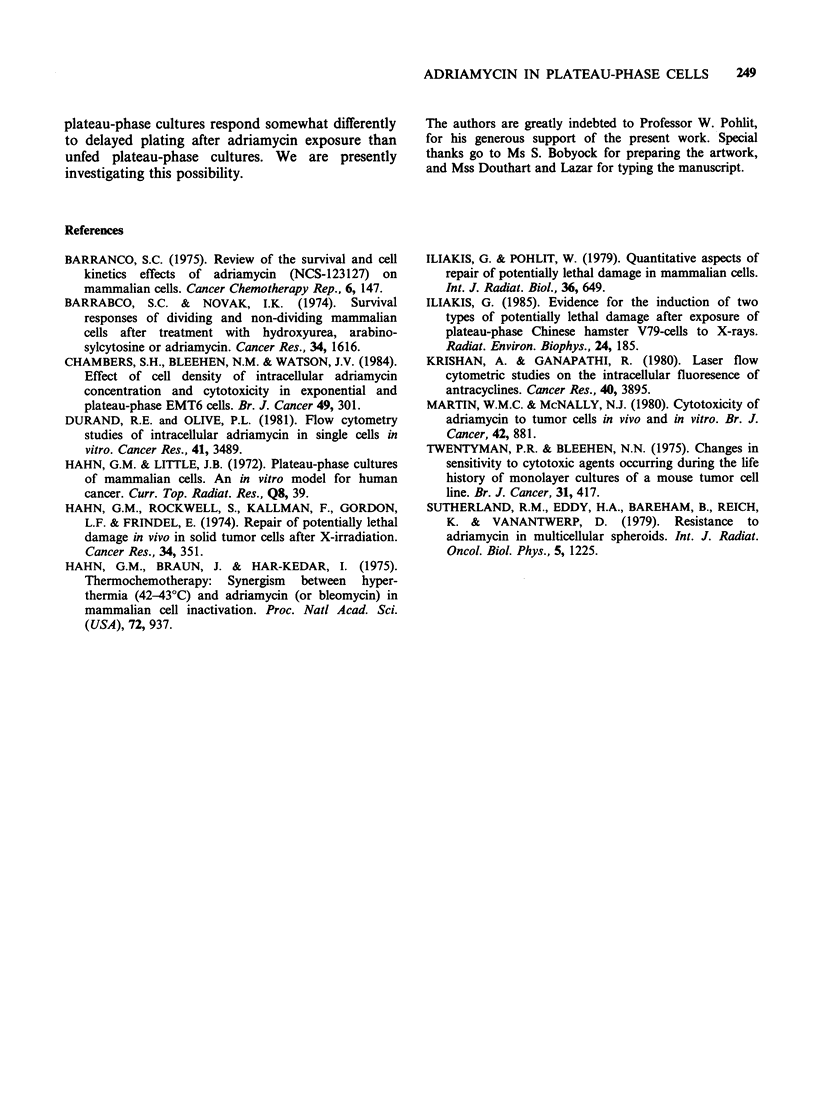


## References

[OCR_00489] Barranco S. C., Novak J. K. (1974). Survival responses of dividing and nondividing mammalian cells after treatment with hydroxyurea, arabinosylcytosine, or adriamycin.. Cancer Res.

[OCR_00495] Chambers S. H., Bleehen N. M., Watson J. V. (1984). Effect of cell density on intracellular adriamycin concentration and cytotoxicity in exponential and plateau phase EMT6 cells.. Br J Cancer.

[OCR_00501] Durand R. E., Olive P. L. (1981). Flow cytometry studies of intracellular adriamycin in single cells in vitro.. Cancer Res.

[OCR_00517] Hahn G. M., Braun J., Har-Kedar I. (1975). Thermochemotherapy: synergism between hyperthermia (42-43 degrees) and adriamycin (of bleomycin) in mammalian cell inactivation.. Proc Natl Acad Sci U S A.

[OCR_00511] Hahn G. M., Rockwell S., Kallman R. F., Gordon L. F., Frindel E. (1974). Repair of potentially lethal damage in vivo in solid tumor cells after x-irradiation.. Cancer Res.

[OCR_00529] Iliakis G. (1985). Evidence for the induction of two types of potentially lethal damage after exposure of plateau phase Chinese hamster V79 cells to gamma-rays.. Radiat Environ Biophys.

[OCR_00524] Iliakis G., Pohlit W. (1979). Quantitative aspects of repair of potentially lethal damage in mammalian cells.. Int J Radiat Biol Relat Stud Phys Chem Med.

[OCR_00535] Krishan A., Ganapathi R. (1980). Laser flow cytometric studies on the intracellular fluorescence of anthracyclines.. Cancer Res.

[OCR_00540] Martin W. M., McNally N. J. (1980). Cytotoxicity of adriamycin to tumour cells in vivo and in vitro.. Br J Cancer.

[OCR_00551] Sutherland R. M., Eddy H. A., Bareham B., Reich K., Vanantwerp D. (1979). Resistance to adriamycin in multicellular spheroids.. Int J Radiat Oncol Biol Phys.

[OCR_00545] Twentyman P. R., Bleehen N. M. (1975). Changes in sensitivity to cytotoxic agents occurring during the life history of monolayer cultures of a mouse tumour cell line.. Br J Cancer.

